# A {Gd_12_Na_6_} Molecular Quadruple-Wheel with a Record Magnetocaloric Effect at Low Magnetic Fields and Temperatures

**DOI:** 10.1021/jacs.3c01610

**Published:** 2023-04-03

**Authors:** Thomais G. Tziotzi, David Gracia, Scott J. Dalgarno, Jürgen Schnack, Marco Evangelisti, Euan K. Brechin, Constantinos J. Milios

**Affiliations:** † Department of Chemistry, University of Crete, Voutes 71003, Herakleion, Greece; ‡ Instituto de Nanociencia y Materiales de Aragón (INMA), CSIC & Universidad de Zaragoza, 50009 Zaragoza, Spain; § Institute of Chemical Sciences, Heriot-Watt University, Riccarton, Edinburgh EH14 4AS, Scotland, U.K.; ∥ Fakultät für Physik, Universität Bielefeld, Postfach 100131, 33501 Bielefeld, Germany; ⊥ EaStCHEM School of Chemistry, The University of Edinburgh, Edinburgh EH9 3FJ, Scotland, U.K.

## Abstract

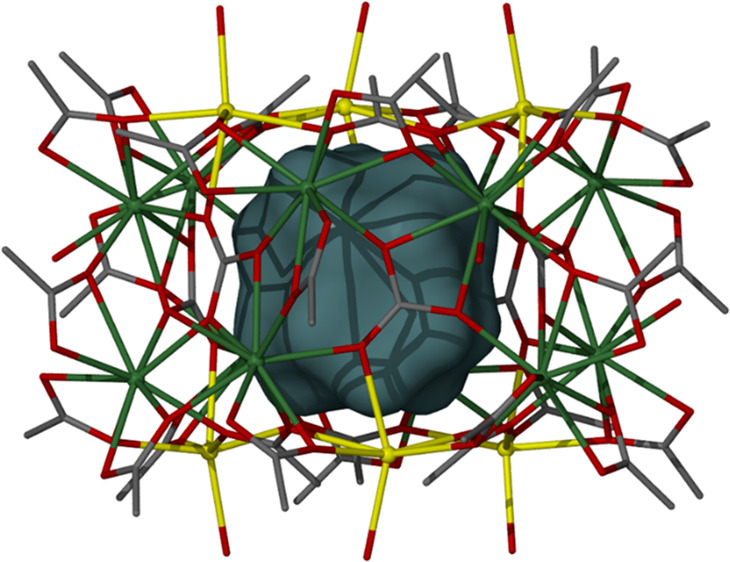

Reaction of Gd(OAc)_3_·4H_2_O, salicylaldehyde and CH_3_ONa in MeCN/MeOH affords [Gd_12_Na_6_(OAc)_25_(HCO_2_)_5_(CO_3_)_6_(H_2_O)_12_]·9H_2_O.0.5MeCN (**1**·9H_2_O.0.5MeCN), whose structure describes a quadruple-wheel consisting of two {Na_3_} and two {Gd_6_} rings. The magnetic properties of **1** reveal very weak antiferromagnetic interactions between the Gd^III^ ions, which give rise to a record magnetocaloric effect at low applied magnetic fields and low temperatures. The magnetic entropy change reaches −Δ*S*_m_= 29.3 J kg^–1^ K^–1^ for full demagnetization from *B* = 1 T at *T* = 0.5 K.

An adiabatic demagnetization refrigerator (ADR) works on the principle of the magnetocaloric effect (MCE). At the heart of the refrigerator, the magnetocaloric material responds to a change of the applied magnetic field (Δ*B*) with a change of adiabatic temperature (Δ*T*_ad_) and magnetic entropy (Δ*S*_m_). The helium shortage and the use of ADRs as cryogenic platforms for quantum technologies and spaceborne experiments have been boosting research on magnetocaloric materials and the development of refrigeration devices.^1^ Modern cryogen-free refrigerators consist of multiple cooling stages, combining both mechanical coolers and ADR modules. Indeed, two or more ADR modules are implemented within the same cryostat to provide continuous cooling, versus the *one-shot* demagnetization cycle provided by a single module,^2^ over a relatively broad temperature span (∼4–0.1 K). For example, the thermal architecture of the LiteBIRD space observatory will include seven ADR modules.^3^ Its cooling stage between ∼1.8 and 0.3 K will consist of two ADR modules, one transiting between these two temperatures and one providing continuous cooling at 0.3 K. This base temperature sets a stringent condition on the choice of magnetocaloric material, that must order magnetically below, or ideally at, this temperature where the MCE is maximized.^4^ Solid-state materials in which Gd is the sole magnetic element do not comply with such a requirement. The few exceptions are all molecular, and include Gd_2_(SO_4_)_3_·8H_2_O,^5,6^ GdCl_3_·6H_2_O,^7^ [{Gd(OAc)_3_(H_2_O)_2_}_2_]·4H_2_O,^8^ Na_9_[Gd(W_5_O_18_)_2_]·35H_2_O and K_12_(GdP_5_W_30_O_110_)·54H_2_O,^9^ [Gd_7_(OH)_6_(thmeH_2_)_5_(thmeH)(tpa)_6_(MeCN)_2_](NO_3_)_2_,^10^ and (iPr_2_NH_2_)_6_[Gd_7_(OH)_3_(CO_3_)_6_(O_2_CtBu)_12_],^11^ which order at/below 0.2 K. Recent results suggest that Ba_2_GdSbO_6_ and Sr_2_GdSbO_6_ might be desirable refrigerants because they do not undergo a phase transition, at least down to 0.4 K.^12^ Unfortunately, the large −Δ*S*_m_ and Δ*T*_ad_ values of materials such as Gd_3_Ga_5_O_12_,^13^ GdF_3_,^14^ Gd(HCOO)_3_,^15^ GdPO_4_,^16^ GdVO_4_,^17^ GdLiF_4_,^18^ and Gd(OH)_3–*x*_F_*x*_^19^ cannot be exploited because the targeted base temperature cannot be reached. Gadolinium is the preferred magnetic element of choice because its large spin and zero orbital angular momentum favor a large MCE.^4^ To attain very low temperatures, traditional paramagnetic “salt pills”, e.g., CPA, FAA, and CMN,^20^ or magnetically frustrated Yb-based compounds, e.g., YbPt_2_Sn,^21^ Yb_3_Ga_5_O_12_,^22^ KBaYb(BO_3_)_2_,^23^ and YbCu_4_Ni,^24^ are the most considered, despite their relatively low MCE. This is because the strength of the applied magnetic field can eventually be increased to compensate for the suboptimal MCE, the drawback being technological complexity and heavier components. Magnetocaloric materials that can be operated at both low and very low temperatures with applied fields produced with permanent magnets (typically 1–2 T) are therefore sought after.^15,25,26^ Here, we report the new molecular nanomagnet [Gd_12_Na_6_(OAc)_25_(HCO_2_)_5_(CO_3_)_6_(H_2_O)_12_]·9H_2_O·0.5MeCN (**1**·9H_2_O·0.5MeCN) that demonstrates an unprecedently large low-field MCE for low and very low temperatures.

Complex **1** crystallizes in the rhombohedral space group *R*-3. Its structure (Figure 1) describes a quadruple-wheel comprising two {Na_3_} and two {Gd_6_} rings held together by a combination of acetate, formate, and carbonate ligands, the latter arising from the fixation of atmospheric CO_2_ (see the Supporting Information (SI) for full details). The metallic skeleton, when viewed in the *ab* plane, is a layered structure comprising a {Na_3_} triangle atop a {Gd_6_} wheel atop a second {Gd_6_} wheel atop a second {Na_3_} triangle. The {Gd_6_)_2_ unit therefore describes a distorted hexagonal prism and the {Na_3_}_2_ moiety a trigonal antiprism (Figure 2). The six lanthanide centers in each {Gd_6_} ring are coplanar, displaying a deviation from the mean plane of ∼0.2 Å. The six carbonate ions sit in the center of the {Gd_6_}_2_ belt and each bond in a η^2^:η^2^:η^2^:μ_5_ fashion to four Gd ions and one Na ion (SI, Figure S1), defining a {Gd_4_Na} pentagon. An alternative view of the metallic core is therefore six edge-sharing {Gd_4_Na(CO_3_)} pentagons fused into a “spherical” cluster. The Gd···Gd distances within each {Gd_4_Na} pentagon are ∼4.0 Å, the Gd···Na distances fall in the range 3.61–3.75 Å. Twelve acetates η^2^:η^1^:μ bridge between the Gd ions in the {Gd_6_}_2_ belt (SI, Figure S2), six along the length of the belt (three in each {Gd_6_} wheel) and six across the breadth of the belt (between the {Gd_6_} wheels). The remaining 18 acetates/formates connect the {Gd_6_} wheels to the {Na_3_} triangles, and they do so in two ways. Six bond in a η^2^:η^2^:μ_4_ fashion to two Gd and two Na ions, lying on the inner rim of the {Na_3_} triangles. It is these carboxylates that show acetate/formate disorder (see the SI for full details); twelve bond in a η^2^:η^1^:μ fashion to one Gd ion and one Na ion, sitting on the exterior of the cage between the {Gd_6_} and {Na_3_} rings.

**Figure 1:**
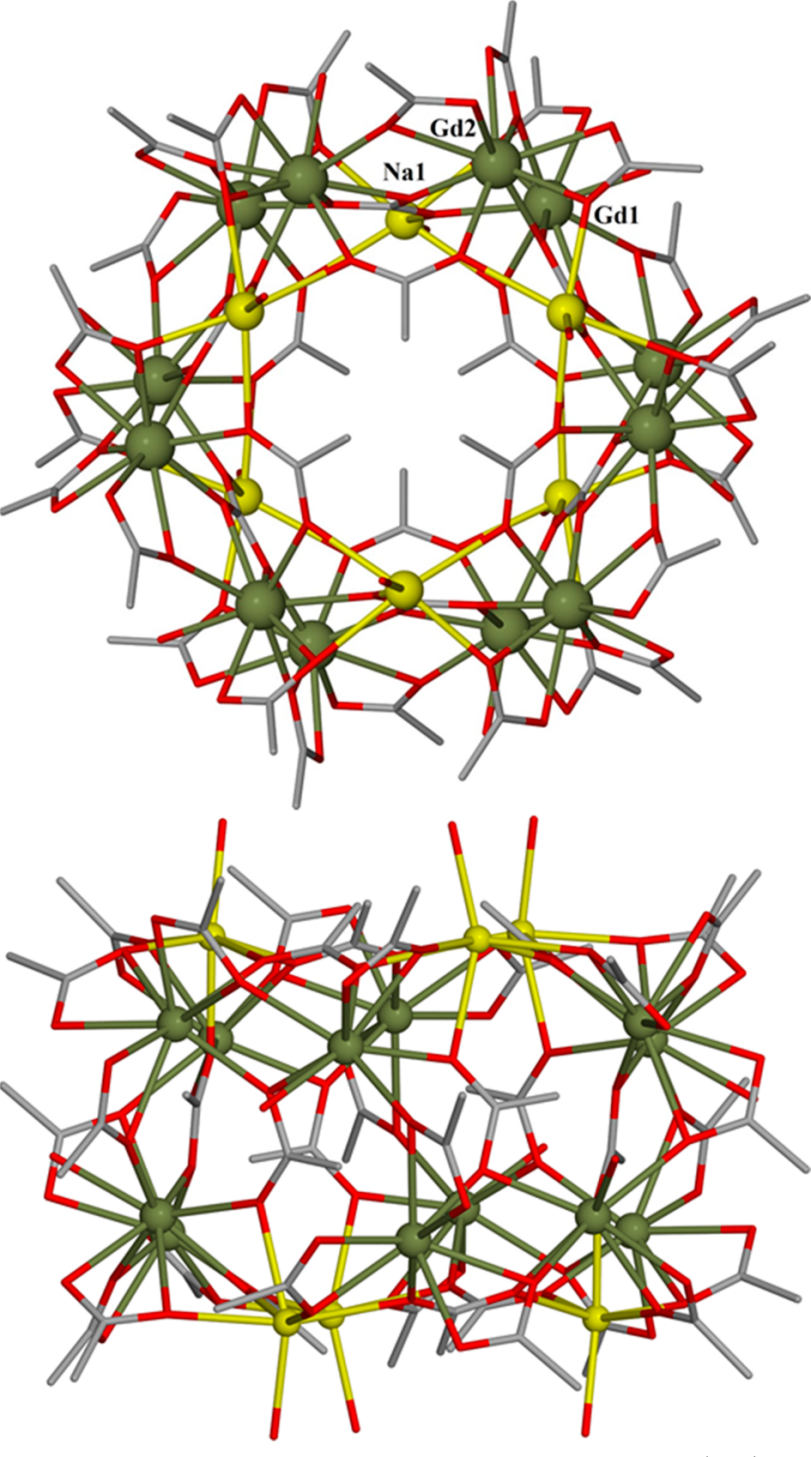
Molecular structure of **1** viewed down the *c* (top) and *a* (bottom) axes. Color code: Gd = olive green, Na = yellow, O = red, C = gray. H atoms and solvent molecules of crystallization are omitted for clarity.

**Figure 2:**
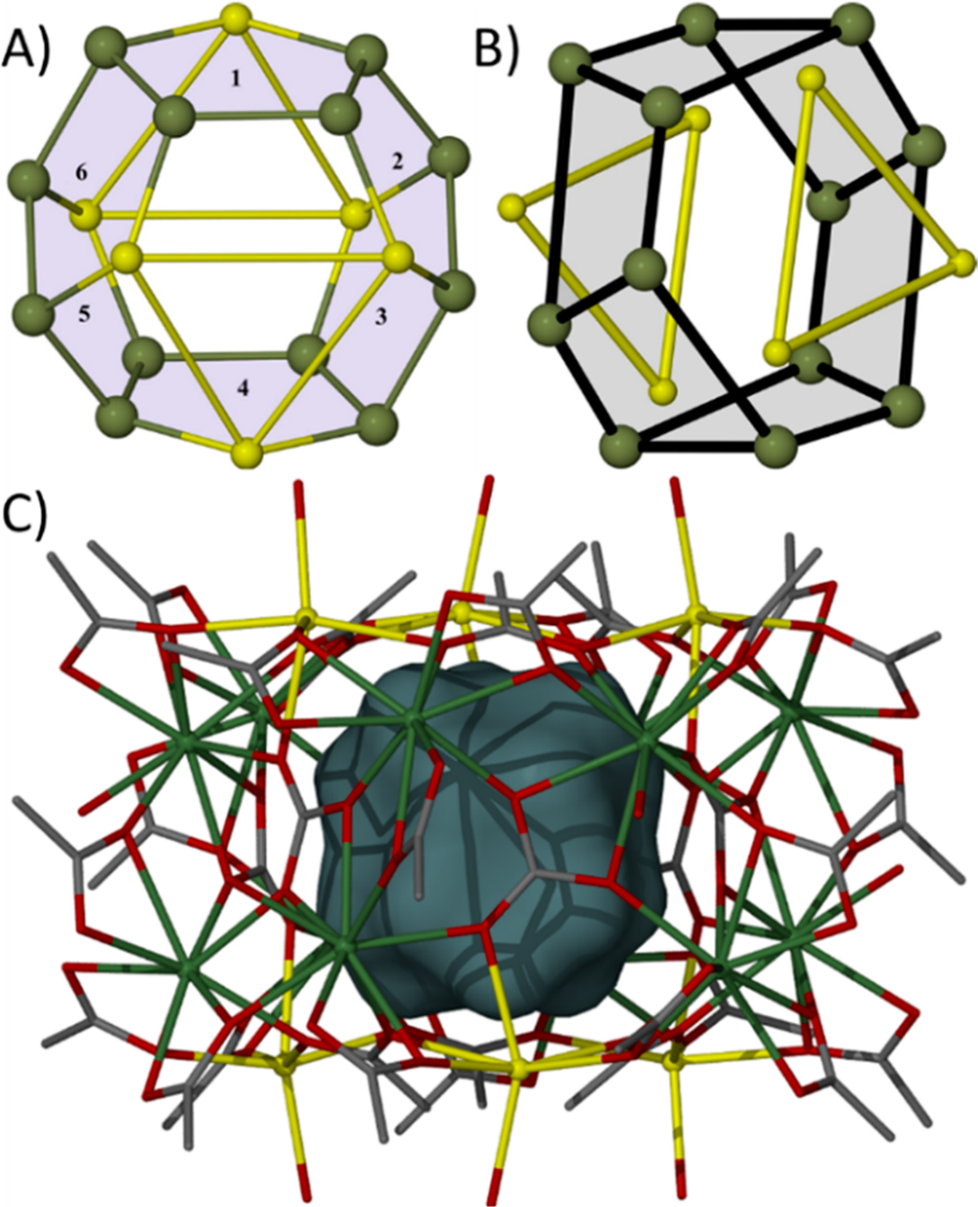
(A) Assembly of the six {Gd_4_Na} pentagons (shaded and numbered 1–6). (B) Metallic skeleton of **1** highlighting the {Gd_6_)_2_ hexagonal prism (shaded) and the {Na_3_}_2_ trigonal antiprism. (C) Structure of **1** highlighting the internal cavity in space-filling representation. Color code: Gd = olive green, Na = yellow, O = red, C = gray. H atoms and solvent molecules of crystallization are omitted for clarity.

The two symmetry inequivalent Gd ions (Gd1, Gd2) are both in {GdO_9_} capped square antiprismatic geometries, the ninth site on Gd1 being occupied by a H_2_O molecule (SI, Figure S3). The symmetry equivalent Na ions (Na1) are six coordinate and in distorted {NaO_6_} octahedral geometries, the sixth site being occupied by a H_2_O molecule. These are H-bonded to acetate O atoms on neighboring clusters, creating an ABC hexagonal close packed arrangement of cages in the lattice (SI, Figure S4). Nearest intercluster Gd···Na and Gd···Gd distances are ∼7.1 and ∼8.1 Å, respectively. Complex **1** has an internal cavity of ∼52 Å^3^ (Figure 2), which is partially (50%) occupied by a MeCN molecule of crystallization which aligns along the *c*-axis of the cell. A search of the Cambridge Structural Database reveals that **1** has a unique structure type.

DC magnetic susceptibility and magnetization measurements (Figure 3, SI, S5) reveal the presence of very weak antiferromagnetic exchange between the Gd^III^ ions. The susceptibility follows a Curie–Weiss law with θ = −0.3 K. The χ*T* product (χ = molar magnetic susceptibility, *T* = temperature) at *T* = 300 K is equal to the Curie constant expected for 12 noninteracting Gd^III^ ions (94.5 cm^3^ K mol^–1^) and is essentially invariant as *T* drops to ∼25 K, wherefrom it decreases to ∼84 cm^3^ K mol^–1^ at 2 K. Magnetization (*M*) data saturate at a value of 84 μ_B_ at *T* = 2 K and a field of *B* = 5 T, as expected for 12 uncorrelated *s* = 7/2 ions. The susceptibility and magnetization data can be simulated using a spin-Hamiltonian  with just one parameter, *J* = −0.01 K with *g* = 2.00, employing a model (SI, Figure S6) that assumes the exchange is mediated through the single O atom acetate bridges and ignores the three atom Gd–O–C–O–Gd bridges.^27,28^

**Figure 3:**
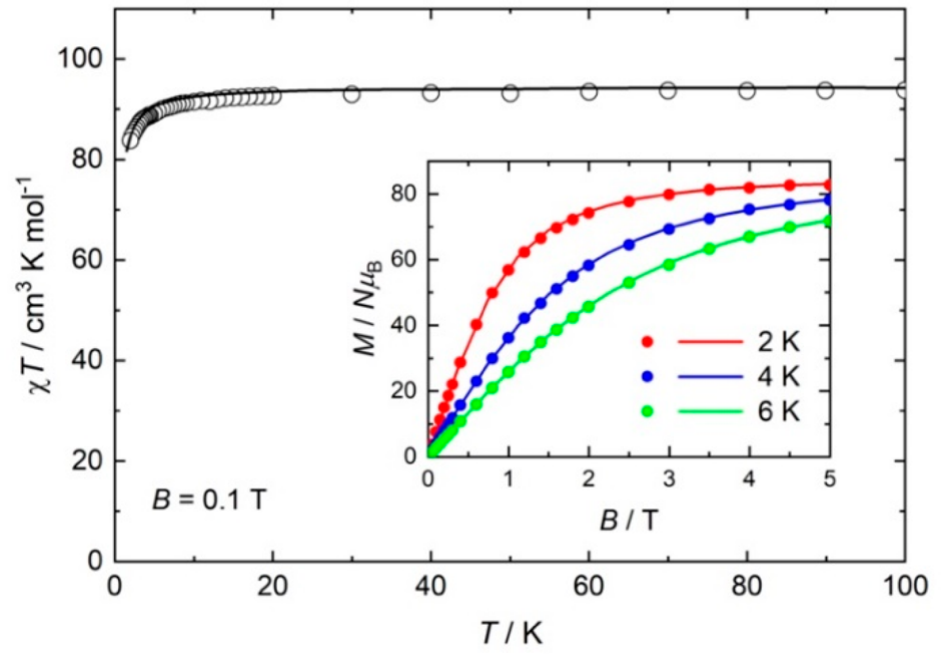
DC magnetic susceptibility at *B* = 0.1 T vs temperature and low-temperature magnetization vs field (inset) for **1**. Experimental data = symbols, theoretical simulations = lines.

The same model can be used to simulate the magnetic contribution to the heat capacity (*c*_p_, Figure 4). All observables, χ, *M*, *c*_p_, can be reproduced nicely, except for the zero-field *c*_p_, where a small internal magnetic field due to dipolar ordering must be assumed. This is a typical procedure for dipolar materials; for large external fields and temperatures, the internal field becomes irrelevant. Assuming an internal field value of *B* = 0.22 T, the Schottky anomaly resulting from the sum of 12 noninteracting Gd^III^ ions per formula unit mimics well the behavior of the zero-field magnetic heat capacity. At high temperatures, *c*_p_ is dominated by the lattice contribution (dashed line in Figure 4), which follows Debye’s law below 5 K, *c*_latt_/*T*^3^ = 3.8 × 10^–2^*R*.

**Figure 4:**
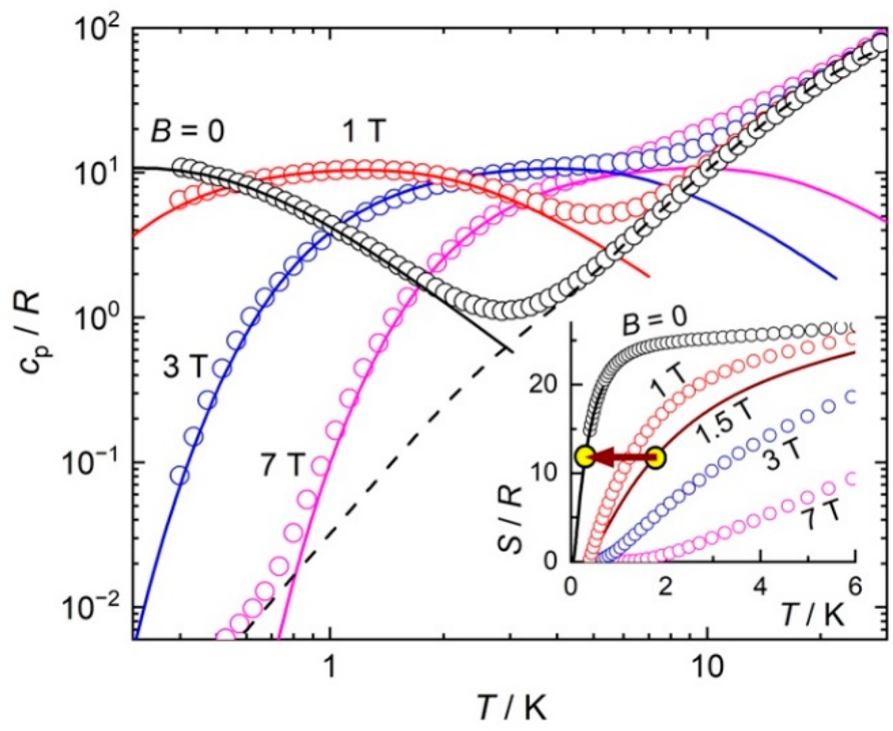
Heat capacity and entropy (inset) vs temperature for selected applied field values for **1**. Experimental data = symbols, theoretical simulations = lines. The arrow highlights the adiabatic demagnetization cooling for the targeted application.

The temperature and field dependencies of the absolute entropy (*S*, Figure 4) are calculated from the heat capacity data, as *S*(*T*,*B*) = ∫_0_^*T*^*c*_*p*_ (*T*′,*B*)/*T*′ d*T*′. Because *c*_p_ was recorded to 0.4 K, the entropy for lower temperatures is obtained from the zero-field Schottky calculation. For fields other than zero, no extrapolation toward absolute zero is needed to obtain *S*. The zero-field entropy data shows that the available magnetic entropy content of **1**, *S*_m_ = 12 × *R* × ln(2*s* + 1) = 24.95R = 46.3 J kg^–1^ K^–1^, is fully attained at very-low temperatures, ca. 2–3 K, as a result of the very weak magnetic interactions. At much higher temperatures, *S*(*T*,*B*) curves increase steadily and tend to overlap because of the nonmagnetic lattice contributions.

Complex **1** meets all the requirements for a large MCE at low and very low temperatures.^4^ The largest possible value of the spin ground state and the negligible anisotropy, combined with very weak magnetic interactions, lead to the absence of phase transitions, at least down to 0.4 K, and to the presence of a large zero-field entropy below 2–3 K. The use of lightweight ligands promotes a large magnetic:nonmagnetic atom ratio and a large magnetic entropy per unit mass.^15^ The MCE figures of merit, Δ*S*_m_ and Δ*T*_ad_, in Figure 5, are obtained as differences between the entropy curves shown in Figure 4, for the magnetic field changes Δ*B* = *B* – 0, i.e., after a full demagnetization from *B*. Δ*S*_m_ is also calculated from the magnetization data in Figure 3 (top inset) and SI, Figure S5, using the Maxwell relation Δ*S*_m_ = ∫[∂*M*/∂*T*]_B_*dB*. Both methods provide identical results.

**Figure 5:**
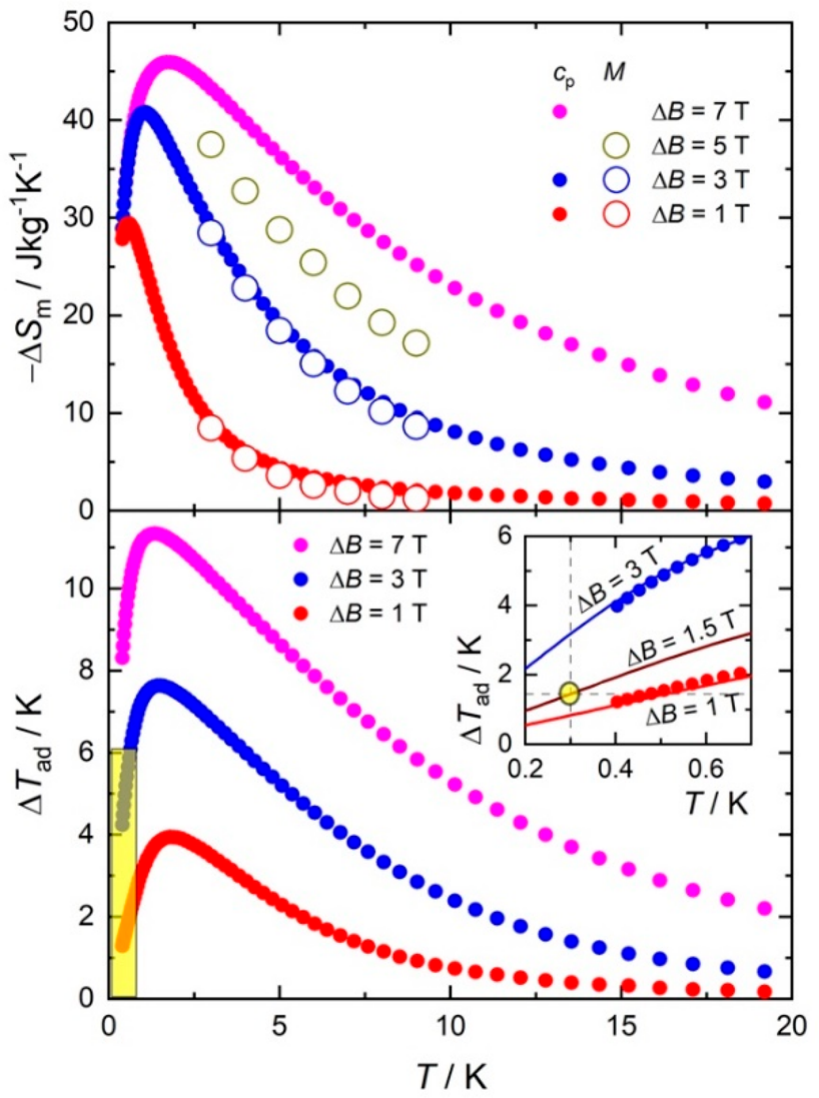
Magnetic entropy change (top) and adiabatic temperature change (bottom) for **1**, calculated from magnetization (empty symbols) and heat capacity (filled symbols) data. Bottom inset: magnification of the low-field/low-temperature (yellow) area. The intersection of the dashed lines (yellow symbol) highlights the targeted temperature (cf. arrow in Figure 4), which is attained for Δ*B* = 1.5 T. Solid lines are theoretical simulations.

The magnetic entropy change reaches the value of −Δ*S*_m_ = 46.0 J kg^–1^ K^–1^, which corresponds to ∼99% of the available entropy at *T* = 1.8 K for the largest applied field change Δ*B* = 7 T (Figure 5). This value is exceptionally large and lags behind only a handful of magnetic refrigerants, all of which are nonmolecular, including Gd(HCOO)_3_,^15^ GdPO_4_,^16^ GdVO_4_,^17^ GdLiF_4_,^18^ Gd(OH)_3–*x*_F_*x*_,^19^ and LiErF_4_.^25^ In contrast to **1**, all of these materials undergo a transition to a magnetically ordered state between ∼0.5 and 0.8 K, which sets the lowest limit of cooling by demagnetization. Where **1** stands apart is in the MCE values attained for far smaller applied field strengths, due to the large entropy that **1** reaches at very low temperatures. For instance, for Δ*B* = 1 T, we observe the unprecedented value of –Δ*S*_m_ = 29.3 J kg^–1^ K^–1^, which corresponds to ∼63% of the available entropy at a remarkably low *T* = 0.5 K (Figure 5). The adiabatic temperature change follows a similar trend. For the largest Δ*B* = 7 T, Δ*T*_ad_ reaches 11.3 K at *T* = 1.3 K. Complex **1** therefore joins the few Gd-based molecular nanomagnets whose Δ*T*_ad_ maximum is known to occur below 2 K for Δ*B* = 7 T, namely [{Gd(OAc)_3_(H_2_O)_2_}_2_]·4H_2_O (Δ*T*_ad_ = 12.6 K at *T* = 1.4 K),^8^ {(μ_3_-CO_3_)_2_[Zn(μ-L)Gd(NO_3_)]_2_}·4CH_3_OH (9.6 at 1.4 K),^29^ [Gd_7_(OH)_6_(thmeH_2_)_5_(thmeH)(tpa)_6_(MeCN)_2_](NO_3_)_2_ (9.4 at 1.8 K),^10^ (^i^Pr_2_NH_2_)_6_[Gd_7_(μ_3_-OH)_3_(CO_3_)_6_(O_2_CtBu)_12_] (9.4 at 1.8 K),^11^ and [Co_3_Gd_3_(H_2_L)_3_(acac)_2_(CH_3_COO)_4_(H_2_O)_2_] (10.7 at 1.5 K).^30^ For field changes as low as Δ*B* = 1 T, **1** has the largest Δ*T*_ad_ = 4.0 K at *T* = 1.8 K.

The MCE for low applied magnetic fields and temperatures deserves special consideration. With θ = −0.3 K, **1** shows a record MCE at the cooling temperatures of the main ADR stage being investigated for future space missions. The 1.8 → 0.3 K cooling by full adiabatic demagnetization is represented by an arrow in Figure 4. This is attained precisely for *B* = 1.5 T, the corresponding entropy curve being calculated with the same model used to simulate the *χ, M* and *c*_*p*_ data. Equivalently, Figure 5 shows that Δ*B* = 1.5 T is needed to target Δ*T*_ad_ = 1.5 K at *T* = 0.3 K. Such a weak applied magnetic field can be produced with permanent magnets, enormously facilitating the implementation of the ADR.

In conclusion, we have synthesized a new molecular magnetic refrigerant, characterized by lightweight ligands that promote extremely weak magnetic correlations between the Gd^III^ centers. This material stands out for its unprecedently large magnetocaloric effect, as observed for temperatures near 0.3 K and low applied magnetic fields, therefore becoming an appealing candidate for refrigeration applications under these conditions.

## Supporting Information Available

Details of X-ray crystallography data collection and structure solution, magnetometry, heat capacity data collection details, and additional figures. (PDF)
