# Supplementary material for: A {Gd12Na6} Molecular Quadruple-Wheel with a Record Magnetocaloric Effect at Low Magnetic Fields and Temperatures
Source: J Am Chem Soc. 2023 Apr 3;145(14):7743–7. doi: 10.1021/jacs.3c01610 (PMC10103126; doi:10.1021/jacs.3c01610)
Supplement: Supplementary file 1 — ja3c01610_si_001.pdf [file ja3c01610_si_001.pdf]

Supplementary Information for

A $\{Gd_{12}Na_6\}$ molecular quadruple-wheel with a record magnetocaloric effect at low magnetic fields and temperatures

Thomais G. Tziotzi,† David Gracia,‡ Scott J. Dalgarno,§ Jürgen Schnack,¶* Marco Evangelisti,‡* Euan K. Brechin,#,* and Constantinos J. Milios†,*

†Department of Chemistry, University of Crete, Voutes 71003, Herakleion, Greece
‡Instituto de Nanociencia y Materiales de Aragón (INMA), CSIC – Universidad de Zaragoza, 50009 Zaragoza, Spain
§Institute of Chemical Sciences, Heriot-Watt University, Riccarton, Edinburgh, EH14 4AS, Scotland, UK
¶ Fakultät für Physik, Universität Bielefeld, Postfach 100131, 33501 Bielefeld, Germany
#EaStCHEM School of Chemistry, The University of Edinburgh, EH9 3FJ, Scotland, UK

Email: jschnack@uni-bielefeld.de; evange@unizar.es; E.Brechin@ed.ac.uk; komil@uoc.gr

Table of Contents

I	General experimental details	S2
II	Synthesis of complex **1**·$9H_2O$·0.5MeCN	S2
III	X-ray diffraction for **1**·$9H_2O$·0.5MeCN	S3
IV	Coordination modes of the CO_2^{3-} and OAc^- ligands present in **1**	S4
V	SHAPE analysis	S5
VI	Crystal packing of **1**·$9H_2O$·0.5MeCN	S6
VII	Magnetization data	S7
VIII	Magnetic exchange interaction model	S8
IX	EDS spectrum of **1**·$9H_2O$·0.5MeCN	S9
X	pXRD for **1**·$9H_2O$·0.5MeCN	S9

I. General experimental details

All reagents and chemicals were purchased from commercially available sources, and all synthetic procedures were performed under aerobic conditions using materials and solvents as received.

Physical Measurements

Elemental analyses (C, H, N) were performed by the University of Ioannina microanalysis service. Variable-temperature, solid-state direct current (dc) magnetic susceptibility data were collected on a Quantum Design MPMS-XL magnetometer. Diamagnetic corrections were applied to the observed paramagnetic susceptibilities using Pascal's constants. Heat capacity measurements were carried out using a Quantum Design PPMS equipped with a ^{3}He cryostat on a thin pressed pellet (ca. 1 mg) of polycrystalline sample, thermalized by ca. 0.2 mg of Apiezon N grease, whose contribution was subtracted by using a phenomenological expression. Powder XRD data were collected on a freshly prepared sample of **1** on a PANanalytical X'Pert Pro MPD diffractometer. Energy-dispersive X-ray Spectroscopy (EDS) measurements were performed on a JEOL Scanning Electron Microscope. FTIR−ATR spectra were recorded on a PerkinElmer FTIR Spectrum BX spectrometer at the University of Crete.

II. Synthesis of complex 1·9H$_2$O·0.5MeCN

Method A

$Gd(OAc)_3 \cdot 4H_2O$ (0.5 mmol, 203 mg), salicylaldehyde (1 mmol, 122 mg) and CH_3ONa (0.5 mmol, 27 mg) were added to a mixture of solvents MeCN/MeOH (3:1), and the resultant pale-yellow solution was left under stirring for 60 minutes during which time no colour change was observed. The solution was then filtered and left undisturbed for slow evaporation, until its volume was reduced to 1/3 of its initial size. During the evaporation process, the solution was filtered three times to remove a colorless amorphous material. Pale-yellow crystals of **1**·9H$_2$O·0.5MeCN formed after 4 days, in ~20% yield. Anal. Calcd (found) for **1**·6H$_2$O: C, 17.01; H, 2.46. Found: C, 16.92, H, 2.27. Main IR data (ATR-FTIR): v= 1545vs, 1448vs, 1403vs, 1369s, 1182m, 1011m, 943m, 894m, 665vs.

Method B

Following the same synthetic procedure as above, the addition of Na_2CO_3 (0.5 mmol) in the reaction mixture leads to a small improvement in the reaction yield, ~30%.

Note that in both cases salicylaldehyde is a reactant but does not appear in the formula of the compound. Reactions which did not include salicylaldehyde, or reactions employing different

aldehydes, did not lead to any crystalline material being isolated. In addition, the formate anions present in the formula are formed upon oxidation of the MeOH/MeO^- species, a procedure common in metal cluster chemistry.[1]

III. X-ray diffraction for 1·9H$_2$O·0.5MeCN

Diffraction data for **1**·9H$_2$O were collected on a Bruker D8 VENTURE diffractometer (University of Crete), equipped with a PHOTION II CPAD detector. **Crystal Data** for $C_{62}H_{123.5}N_{0.5}Gd_{12}Na_6O_{99}$ (M =4485.05 g/mol): trigonal, space group R-3 (no. 148), a = 18.5898(4) Å, c = 35.2955(12) Å, V = 10563.3(6) Å^3, Z = 3, T = 210.0 K, μ(MoKα) = 5.689 mm^{-1}, $Dcalc$ = 2.115 g/cm^3, 25870 reflections measured (5.264° ≤ 2Θ ≤ 58.358°), 6340 unique (R_{int} = 0.0332, R_{sigma} = 0.0337) which were used in all calculations. The final R_1 was 0.0340 (I > 2σ(I)) and wR_2 was 0.0873 (all data). The structure of **1** was solved with the SHELXT structure solution program using Intrinsic Phasing and refined with the SHELXL refinement package using Least Squares minimization.[2-4] The ligands that bridge the Na and Gd ions at the triangular Na_3 faces are disordered such that the methyl of the acetate is present at 1/6th occupancy. Modelling at this occupancy is consistent with the refined value and improved agreement indices accordingly. The cage encapsulates diffuse electron density which is suggestive of disordered, partial occupancy MeCN of crystallization. This was removed using a solvent mask in Olex 2 as it could not be modelled satisfactorily. Full details can be found in the CIF file with CCDC reference number 2216494.

References

1. See for example: Milios, C. J.; Vinslava, A.; Wernsdorfer, W.; Prescimone, A.; Wood, P. A.; Parsons, S.; Perlepes, S. P.; Christou, G.; Brechin, E. K. Spin Switching via Targeted Structural Distortion. *J. Am. Chem. Soc.* **2007**, *129*, 6547-6561; Biswas, B.; Khanra, S.; Weyhermüller, T.; Chaudhuri, P. A one-pot synthesis of a paramagnetic high-nuclearity nickel(II) cluster: an octadecanuclear $Ni^{II}_{16}Na^{I}_{2}$ metal aggregate *Chem.Commun*. **2007**, 1059-1061.
2. Dolomanov, O.V.; Bourhis, L. J.; Gildea, R. J.; Howard, J. A. K.: Puschmann, H. OLEX2: A Complete Structure Solution, Refinement and Analysis Program. *J. Appl. Cryst*. **2009**, *42*, 339-341.
3. Sheldrick, G. M. SHELXT - Integrated space-group and crystal-structure determination. *Acta Cryst*. **2015**, *A71*, 3-8.
4. Sheldrick, G.M. Crystal structure refinement with SHELXL. *Acta Cryst*. **2015**, *C71*, 3-8.

IV. Coordination modes of the CO_3^{2-} and OAc^- ligands present in 1

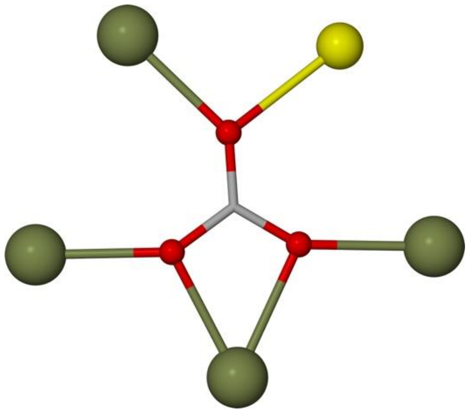

Figure S1. Schematic highlighting the CO_3^{2-} bonding mode in the Gd_4Na pentagons. Color code as Figure 1.

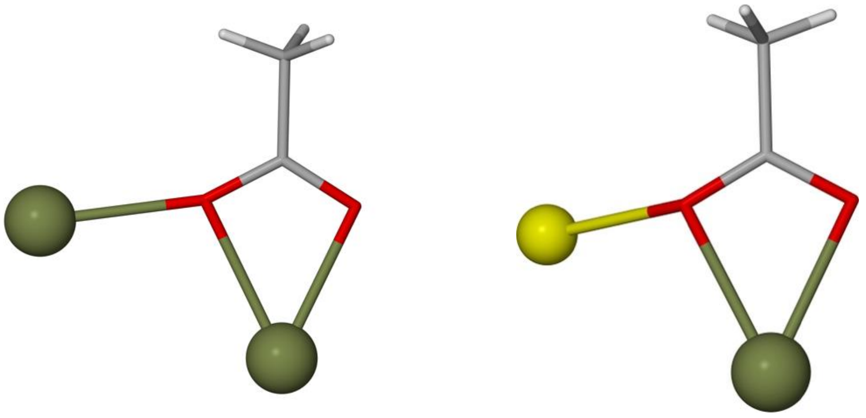

Figure S2. Schematic highlighting the OAc^- bonding mode in the $\{Gd_6\}_2$ belt (left) and the OAc^-/O_2CH^- bonding mode in the $\{Gd_2Na_2\}$ upper and lowers rims of the cluster. Color code as Figure 1.

V. SHAPE analysis for **1**·9H_2O·0.5MeCN

Table S1. SHAPE analysis for **1**·9H_2O·0.5MeCN

Structure [ML_9]	Symmetry	Geometry	Gd1	Gd2
EP-9	D_{9h}	Enneagon	35.141	32.529
OPY-9	C_{8v}	Octagonal pyramid	23.130	23.495
HBPY-9	D_{7h}	Heptagonal bipyramid	18.154	16.328
JTC-9	C_{3v}	Johnson triangular cupola J3	16.090	12.342
JCCU-9	C_{4v}	Capped cube J8	10.635	8.895
CCU-9	C_{4v}	Spherical-relaxed capped cube	8.904	7.957
JCSAPR-9	C_{4v}	Capped square antiprism J10	3.600	3.444
**CSAPR-9**	**C_{4v}**	**Spherical capped square antiprism**	**2.452**	**2.381**
JTCTPR-9	D_{3h}	Tricapped trigonal prism J51	4.543	3.615
TCTPR-9	D_{3h}	Spherical tricapped trigonal prism	3.005	3.127

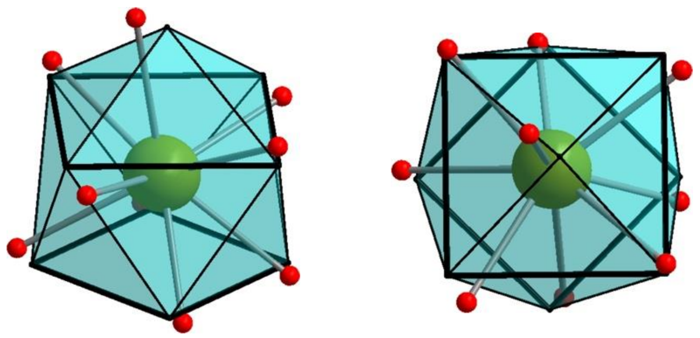

Figure S3. The coordination geometries of Gd1 (left) and Gd2 (right).

VI. Crystal packing of 1·9H_2O·0.5MeCN

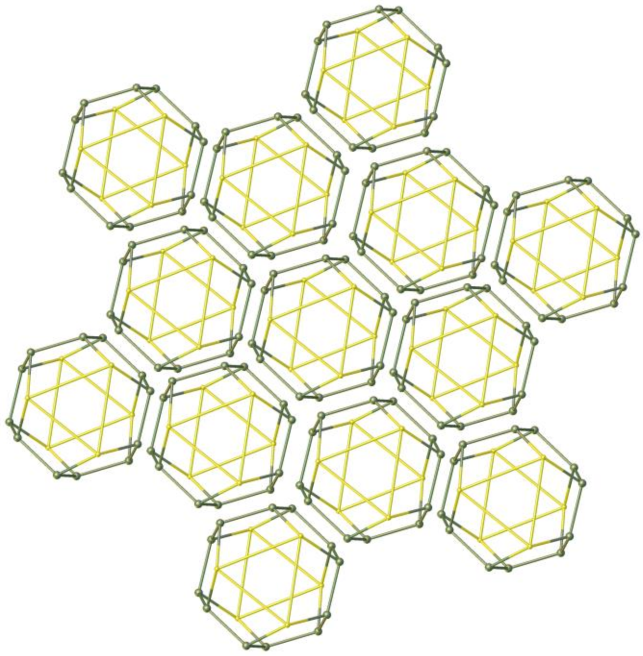

Figure S4. Extended structure showing the ABC hexagonal close packed arrangement of **1** in the lattice.

VII. Magnetization data

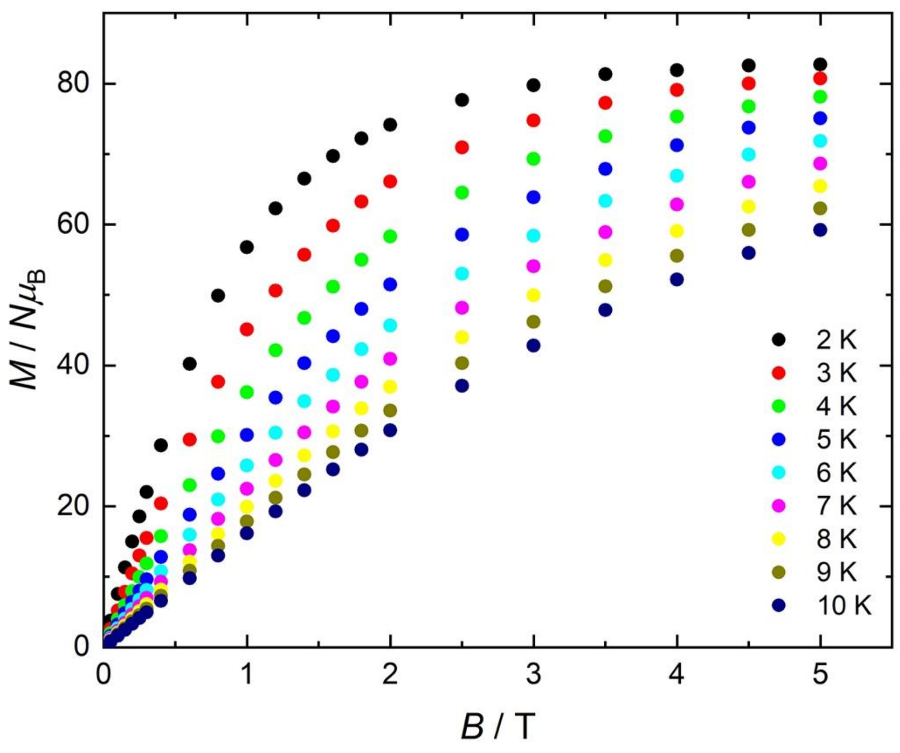

Figure S5. Isothermal magnetization vs. applied magnetic field for selected temperatures, as labelled, for **1**.

VIII. Magnetic exchange interaction model

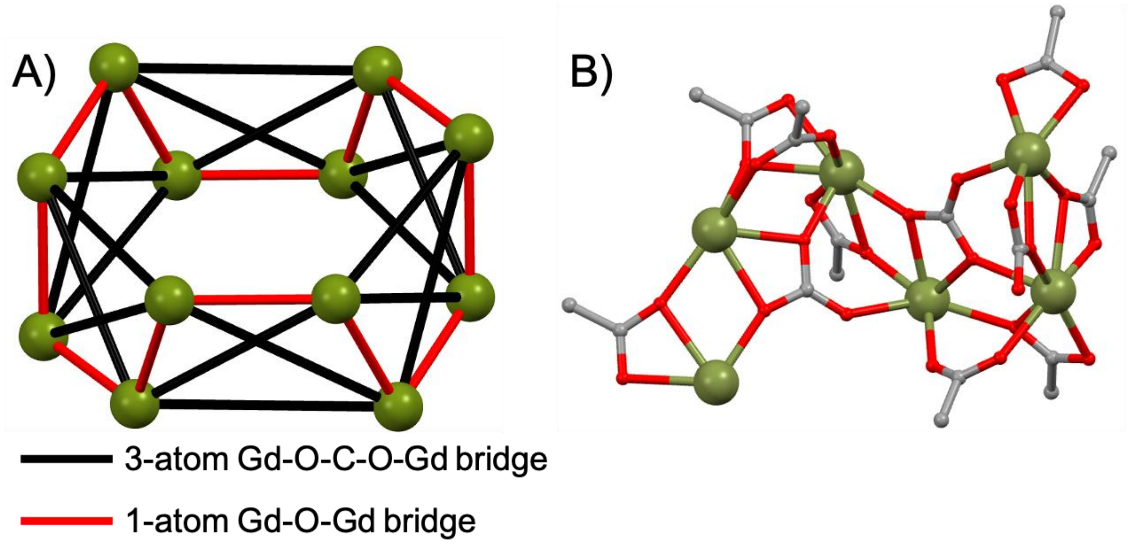

Figure S6. (A) Schematic of the exchange interaction model used to simulate the dc susceptibility, magnetization and heat capacity data. The model employs just one exchange interaction which assumes the exchange through the one-atom Gd-O-Gd bridge (red line) is much larger than the exchange through the three atom Gd-O-C-O-Gd bridge (black lines). (B) A selected portion of the structure of **1** highlighting the magnetic connectivity repeat unit. Color code as Figure 1.

IX. EDS spectrum of 1·9H_2O·0.5MeCN

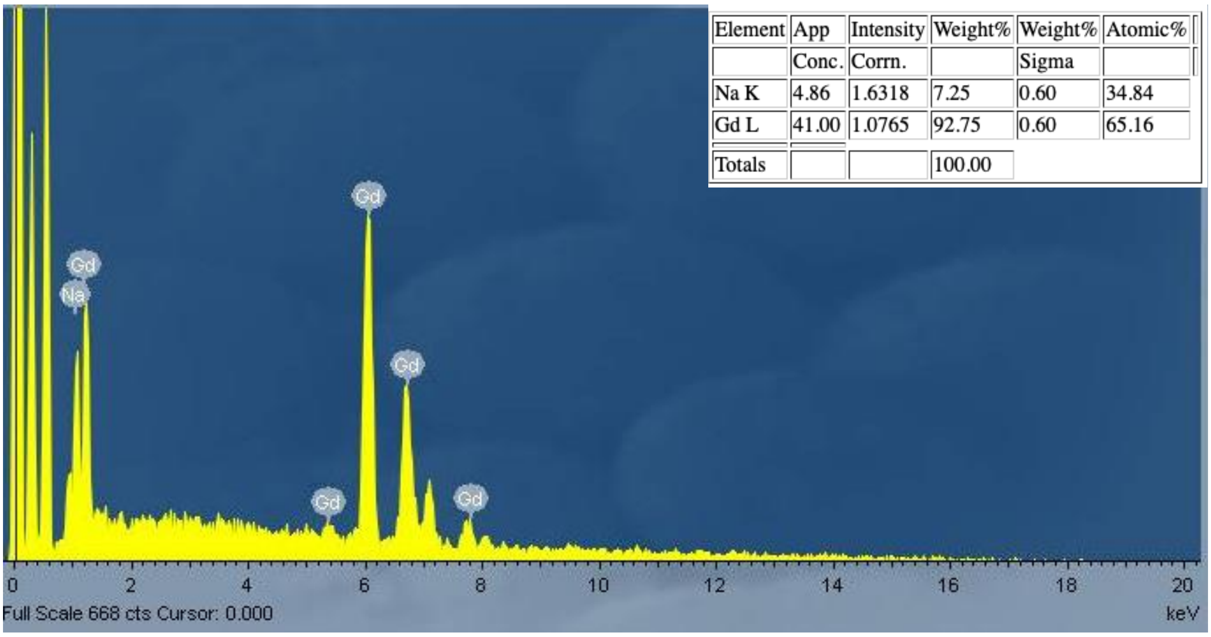

Figure S7. The EDS spectrum of **1**·9H_2O·0.5MeCN. The experimental Gd:Na ratio of 1.87 is in excellent agreement with the theoretical value of 2, as expected from the crystal structure of **1**·9H_2O·0.5MeCN.

X. pXRD of 1·9H_2O·0.5MeCN

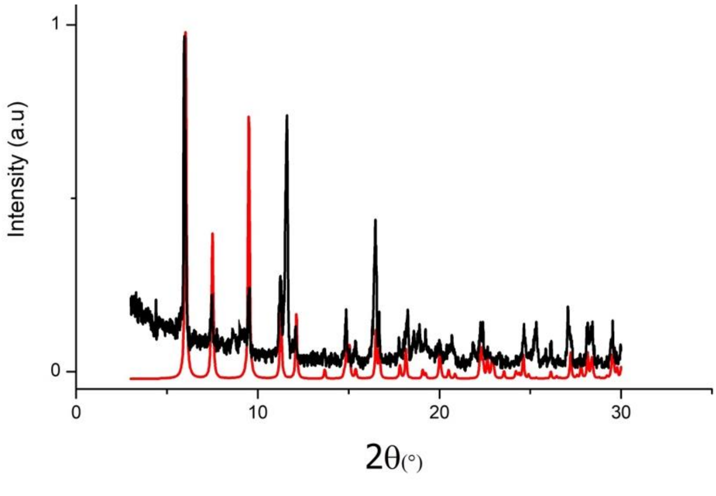

Figure S8. Powder XRD patterns for **1·9H_2O·0.5MeCN** (black line) and its simulated pXRD diagram (red line). The differences in peaks intensity are due to solvent loss and preferred crystal orientation.
